# Single Versus Double Drainage After Modified Radical Mastectomy: A Systematic Review and Meta-Analysis of Randomized Controlled Trials

**DOI:** 10.3390/jcm15218391

**Published:** 2026-10-28

**Authors:** Ahmed Abu-Zaid, Yara S. Alseddiqi, Mohammad M. Sarkhouh, Laila Alkanderi, Fahhad Nafea Alajmi, Hussain A. Alshammari, Fatemah A. Ahmad, Othman M. Alrashidi, Ali Alshooli, Ali J. Alrashidi, Mohammad M. Jassem, Layal T. Alwazzan, Mohammad F. Al-Mutairi, Abdullah M. Alharran

**Affiliations:** 1 Department of Biochemistry and Molecular Medicine, College of Medicine, Alfaisal University, Riyadh 11533, Saudi Arabia; 2 Royal College of Surgeons in Ireland, D02 YN77 Dublin, Ireland; 3 University of Glasgow, Glasgow G12 8QQ, UK; 4 College of Medicine and Medical Sciences, Arabian Gulf University, Manama 22979, Bahrain; 5 Faculty of Medicine, Alexandria University, Alexandria 21531, Egypt; 6 Ministry of Health, Kuwait City 13001, Kuwait; 7 Royal College of Surgeons in Ireland, Busaiteen 228, Bahrain

**Keywords:** breast cancer, mastectomy, surgical drainage, seroma, postoperative complications, clinical trial, meta-analysis

## Abstract

**Background:** Modified radical mastectomy (MRM) with axillary lymph-node dissection frequently leads to postoperative seroma formation, prompting the routine use of closed-suction drainage. While standard practice traditionally involves placing two drains, one axillary and one pectoral/sub-flap, this approach increases patient discomfort and limits mobility. A single-drain strategy may alleviate this burden, but concerns persist that inadequate drainage could increase the risk of seroma. **Methods:** A comprehensive search across five databases was conducted up to 10 August 2026 for randomized controlled trials (RCTs) comparing single axillary drainage with double (axillary plus pectoral/sub-flap) drainage after MRM. The primary outcome was postoperative seroma formation. Risk ratios (RR) and mean differences (MD) with 95% confidence intervals (CI) were pooled using a random-effects model, and the certainty of evidence was rated using the GRADE approach. **Results:** Nine RCTs involving 856 patients were included. There was no statistically significant difference between single and double drainage regarding the incidence of postoperative seroma (RR: 1.13, 95% CI [0.86, 1.47]; *p* = 0.37; low certainty), corresponding to 30 more seromas per 1000 patients (95% CI: 33 fewer to 110 more). Also, the strategies yielded comparable outcomes for the volume of aspirated seroma (MD: 0.19 mL; *p* = 0.97) and the number of post-removal aspirations (MD: 0.13; *p* = 0.32). Total drain output, drain duration, and the risk of complications, including hematoma (RR: 2.01; *p* = 0.27), flap necrosis (RR: 0.72; *p* = 0.36), and surgical site infection (RR: 0.75; *p* = 0.60), did not differ significantly between the two groups. **Conclusions:** Single axillary drainage after MRM was not associated with significant differences in postoperative seroma formation, aspirated seroma volume, number of seroma aspirations, total drain output, drain duration, hematoma, flap necrosis, or surgical-site infection compared with double axillary plus pectoral/sub-flap drainage. However, the certainty of evidence was low for most outcomes and the confidence intervals were compatible with clinically relevant differences, so equivalence cannot be concluded; adequately powered trials with standardized seroma definitions and patient-centered outcomes are needed.

## 1. Introduction

Breast cancer remains one of the most common malignancies worldwide, and surgery remains critical for its treatment [1]. Although breast-conserving surgery is increasingly used, mastectomy remains necessary depending on tumor extent, patient preference, or oncologic considerations [2]. Modified radical mastectomy (MRM), which combines removal of the breast with axillary lymph-node dissection, creates a raw surface and dead space in the axilla and beneath the skin flaps [3]. Disruption of the lymphatic channels and tissue planes can lead to fluid accumulation; thus, seroma is one of the most common complications following mastectomy [4,5]. Seroma can cause pain and wound tension, which can delay wound healing, require repeated aspirations and clinic visits, and increase the risk of infection and delayed recovery [6,7].

Hence, closed-suction drainage has been adopted in routine management after mastectomy and axillary dissection to decrease postoperative seroma formation [8]. Drains evacuate blood and serous or lymphatic fluid and help keep the skin flaps apposed to the chest wall [9]. The current standard practice is to place two drains: one axillary drain for lymphatic drainage and a second drain under the pectoral or mastectomy flap [6]. This technique is widely used but can increase discomfort, limit mobility, prolong drain-related care, and add to the burdens and costs of care [9]. However, drainage practices remain heterogeneous, with different suction pressures, durations, and criteria for drain removal [10].

These concerns have led to interest in discarding the pectoral or sub-flap drain and using a single axillary drain instead, as if one axillary drain is sufficiently evacuative, a second drain may be a burden without meaningful clinical benefit [11]. A single-drain strategy may reduce patient discomfort, facilitate mobilization, facilitate discharge, and potentially decrease total healthcare costs [12]. However, this strategy raises concerns about insufficient drainage of the mastectomy bed that may lead to increased seroma formation, aspiration needs, or wound-related complications [13]. This question has been evaluated in multiple randomized controlled trials (RCTs) [11,12,13,14,15,16,17,18,19], yet the optimal drainage strategy remains uncertain.

Drainage after breast cancer surgery has been addressed by several systematic reviews and meta-analyses, but these have examined whether a drain should be used at all [20], when drains should be removed [10,21], the level of suction applied [22], or seroma-prevention strategies more broadly [23]. The number of drains, by contrast, has been discussed only narratively [3,24], largely on the basis of two trials published in 1992 [25,26], and, to our knowledge, the randomized evidence comparing single with double drainage after MRM has not previously been systematically identified, critically appraised, and pooled.

Given the frequency of seroma and the burden of postoperative drainage, determining whether a second pectoral or sub-flap drain is necessary is clinically significant. Therefore, this systematic review and meta-analysis aimed to compare single axillary drainage with double axillary plus pectoral/sub-flap drainage after MRM for breast cancer, with explicit attention to how the included trials defined and assessed seroma and managed their drains.

## 2. Methods and Materials

### 2.1. Protocol Registration

This systematic review and meta-analysis were prospectively registered with the International Prospective Register of Systematic Reviews (PROSPERO) (registration number: CRD420261493390). The review was conducted and reported in accordance with the Preferred Reporting Items for Systematic Reviews and Meta-Analyses (PRISMA) guidelines [27]. The completed PRISMA checklist is provided in Table S1.

### 2.2. Data Sources and Search Strategy

A.A.Z. and Y.S.A. conducted a systematic literature search across five electronic databases (PubMed, Web of Science, the Cochrane Central Register of Controlled Trials (CENTRAL), Scopus, and Embase) on 10 August 2026. The search strategy combined controlled vocabulary and free-text terms related to mastectomy, surgical drainage, and single- versus double-drain placement. No restrictions based on country, language, or publication status were applied. The complete search strategy and search output for each database are provided in Table S2. The reference lists of the included studies and relevant reviews were also manually screened to identify potentially eligible trials that the database searches may have missed.

### 2.3. Eligibility Criteria

We included RCTs according to the following population, intervention, comparator, and outcomes (PICO) framework:

- Population: Adult patients (>18 years) with breast cancer undergoing MRM, defined as total (simple) mastectomy with preservation of the pectoralis major muscle combined with axillary lymph node dissection (ALND), irrespective of whether the trial authors labelled the procedure as MRM or as total mastectomy with axillary dissection. Trials of breast-conserving surgery, mastectomy with sentinel lymph node biopsy only, mastectomy with immediate reconstruction, or ALND alone were not eligible.
- Intervention: Placement of a single postoperative closed-suction drain positioned in, or directed toward, the axilla.
- Comparator: Placement of two postoperative closed-suction drains, consisting of one axillary drain and an additional pectoral, sub-flap, or mastectomy-bed drain.
- Outcomes: The primary outcome was postoperative seroma formation, as defined by each trial. Secondary outcomes included total drain output or drainage volume, drain duration or time to drain removal, volume of aspirated seroma, number of post-removal seroma aspirations, flap necrosis, hematoma, and wound infection or surgical-site infection (SSI). Pain, patient comfort, and other patient-reported or functional outcomes were summarized narratively because they were not reported consistently enough to permit pooling.

Studies were required to report at least one eligible outcome. We excluded non-randomized studies, observational studies, case reports, case series, conference abstracts without sufficient outcome data, protocols, reviews, and editorials.

### 2.4. Study Selection

Study selection was conducted using Covidence software (Veritas Health Innovation, Melbourne, Australia). Records retrieved from the databases and through citation searching were imported into Covidence, where duplicate records were removed automatically (with additional duplicates identified manually) and records flagged as ineligible by the software’s automation tools were removed before manual screening; these numbers are reported separately in the PRISMA flow diagram. At least two reviewers (M.M.S. and L.A.) independently screened the remaining records in two stages. Titles and abstracts were initially screened to exclude clearly irrelevant records, after which the full texts of potentially eligible articles were assessed against the prespecified eligibility criteria. Disagreements regarding study eligibility were resolved through discussion and consensus. When consensus could not be reached, a third reviewer was consulted.

### 2.5. Data Extraction

A standardized Microsoft Excel extraction form was developed and pilot-tested using a subset of the eligible studies. At least two reviewers (F.N.A. and H.A.A.) independently extracted data, and discrepancies were resolved through discussion or consultation with a third reviewer. Extracted study characteristics included the first author, publication year, country, study design, type of mastectomy and axillary procedure (including the reported extent of axillary dissection), use of flap fixation or quilting sutures, drain type and suction system, total sample size, details and anatomical placement of the single- and double-drain strategies, criteria for drain removal and how drain duration was defined in the double-drain arm (time to removal of the last drain versus per-drain reporting), eligibility criteria, primary outcome, and duration of follow-up. For the primary outcome, we additionally recorded the definition of seroma used by each trial, the method of assessment (clinical examination, ultrasonography, or need for aspiration), the assessment schedule, the indication or threshold for aspiration, and whether the reported counts referred to any seroma or only to seroma requiring intervention. Extracted participant characteristics included the number of participants in each group, age, body mass index (BMI), diabetes mellitus, and, when reported, smoking status. The following outcomes were extracted: seroma formation, total drain output or drainage volume, drain duration or time to drain removal, volume of aspirated seroma, number of post-removal seroma aspirations, flap necrosis, hematoma, and wound infection or SSI. For the volume of aspirated seroma and the number of aspirations, we recorded whether the reported means referred to all randomized patients or only to patients who developed seroma. Pain, patient comfort, functional outcomes, length of hospital stay, and costs were extracted for narrative synthesis.

For continuous outcomes, we extracted the mean, standard deviation (SD), and number of participants analyzed in each group. For dichotomous outcomes, we extracted the number of participants experiencing the event and the total number of participants analyzed in each group. When required numerical information was not directly reported, values were derived from the available study data using standard methods recommended in the Cochrane Handbook [28]. 

### 2.6. Risk of Bias and Certainty of Evidence

At least two reviewers (F.A.A. and O.M.A.) independently assessed the risk of bias in each included randomized trial using the revised Cochrane Risk of Bias 2 tool (RoB 2) [29]. Assessment considered the following domains: bias arising from the randomization process; bias due to deviations from the intended interventions; bias due to missing outcome data; bias in the measurement of the outcome; and bias in the selection of the reported result. Each domain and the overall risk of bias were classified as low risk, some concerns, or high risk.

The certainty of evidence for each major pooled outcome was evaluated using the Grading of Recommendations Assessment, Development and Evaluation (GRADE) framework [30]. Evidence from randomized trials initially received a high-certainty rating and was downgraded, when appropriate, for risk of bias, inconsistency, indirectness, imprecision, or publication bias. The certainty of evidence was categorized as high, moderate, low, or very low, and the rationale for each judgment was documented. Disagreements were resolved through discussion and consensus.

Imprecision was judged from the 95% CI of the pooled estimate and the optimal information size (OIS), following GRADE guidance [30]. For dichotomous outcomes, a 25% relative reduction or increase in risk (RR of 0.75 and 1.25) was taken as the threshold for an appreciable effect, and the OIS was calculated as the total sample size required to detect a 25% relative risk reduction with 80% power and a two-sided α of 0.05, given the pooled control-group risk. Imprecision was rated as serious when the 95% CI crossed one of these thresholds or when the pooled sample size was smaller than the OIS, and as very serious when the 95% CI included both appreciable benefit and appreciable harm and the OIS was not met. For continuous outcomes, we used the following thresholds for a clinically important difference: 100 mL for total drain output, 1 day for drain duration, 50 mL for the volume of aspirated seroma, and 1 aspiration for the number of post-removal aspirations; imprecision was rated as not serious when the 95% CI lay entirely within these thresholds. Anticipated absolute effects were calculated by applying the pooled RR and its 95% CI to the pooled control-group (double-drain) risk across the trials contributing to each outcome.

### 2.7. Meta-Analysis

All statistical analyses were performed using Stata/SE version 19 (StataCorp LLC, College Station, TX, USA). Continuous outcomes were analyzed using mean differences (MDs) because they were reported in comparable units across studies. Dichotomous outcomes were analyzed using risk ratios (RRs). All effect estimates were reported with corresponding 95% confidence intervals (CIs). Random-effects models using restricted maximum likelihood (REML) estimation were used as the primary analytical approach because clinical and methodological heterogeneity was anticipated across studies, including differences in surgical technique, drain positioning, drain removal criteria, and postoperative management. For trials with zero events in one or both arms, a continuity correction of 0.5 was added to all cells of the trial’s 2 × 2 table (the default in Stata); trials with zero events in both arms (one trial each for hematoma and SSI) were retained and therefore contributed an RR of 1.00 with a very wide CI and a small weight to the pooled estimate, which, if anything, pulls the pooled RR toward the null. For the volume of aspirated seroma and the number of aspirations, data were pooled as reported by each trial, noting that the denominators differed between trials (all randomized patients in some trials and patients who developed seroma in others).

Statistical heterogeneity was assessed using Cochran’s Q test and quantified using the I^2^ statistic. Heterogeneity was considered substantial when the Q-test *p*-value was <0.10 or the I^2^ value exceeded 50%. When substantial heterogeneity was detected, and at least three studies were available, leave-one-out sensitivity analyses were conducted by sequentially excluding each study to assess the robustness of the pooled estimate and identify potentially influential studies. Finally, publication bias and small-study effects were not formally assessed using funnel plots or statistical tests for asymmetry because all meta-analyzed outcomes included fewer than 10 studies [31].

## 3. Results

### 3.1. Search Results and Study Selection

**Figure 1:**
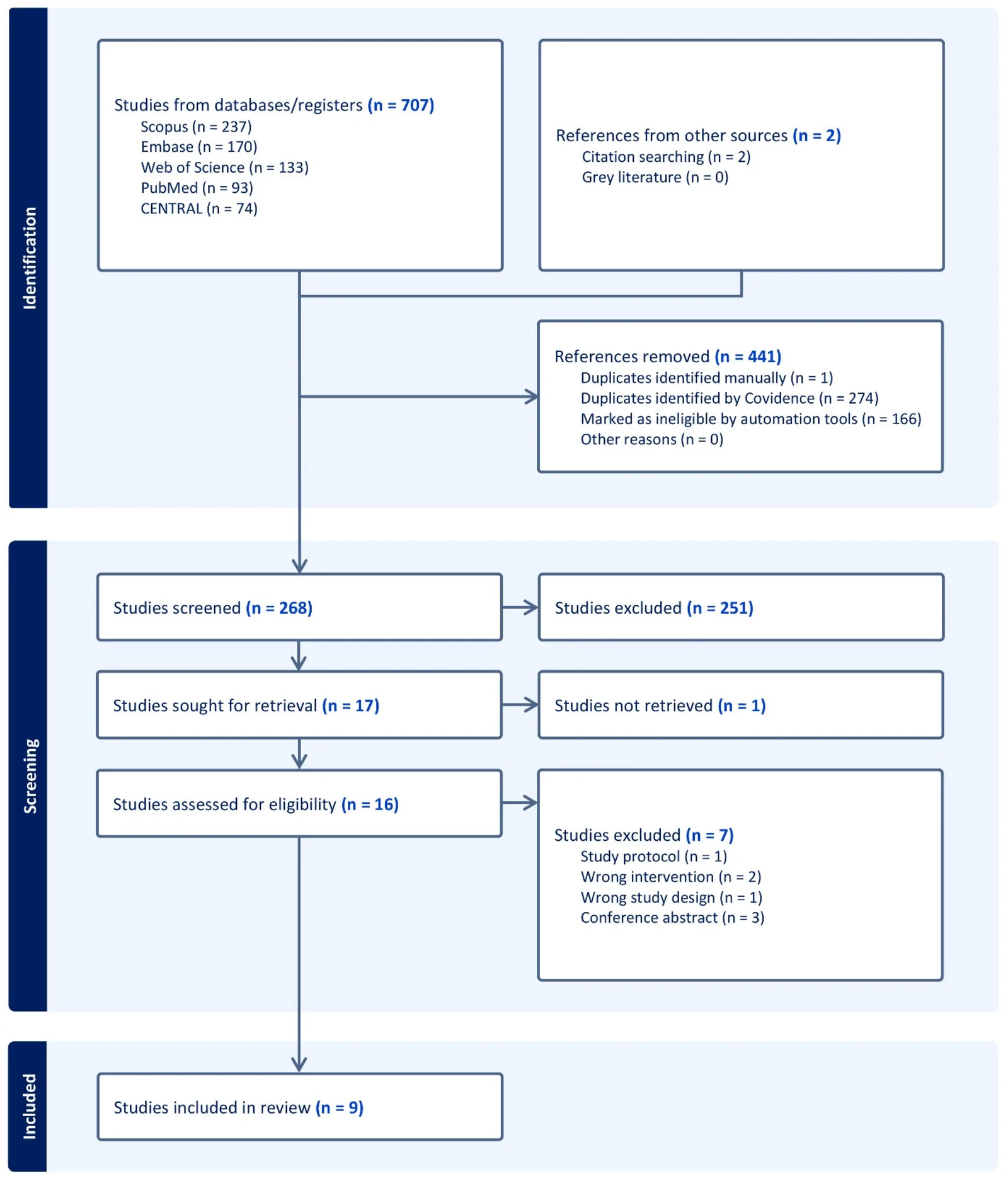
PRISMA flow chart of the study screening and selection process.

A total of 709 records were identified: 707 through database searching (Scopus, n = 237; Embase, n = 170; Web of Science, n = 133; PubMed, n = 93; CENTRAL, n = 74) and 2 through citation searching of the reference lists of included studies and relevant reviews. Before screening, 441 records were removed: 275 duplicates (274 identified by Covidence and 1 identified manually), 166 records marked as ineligible by automation tools, and none for other reasons. The remaining 268 records were screened, of which 251 were excluded. Seventeen reports were sought for retrieval, and one could not be retrieved. Sixteen full-text reports were assessed for eligibility, of which seven were excluded for various reasons (Table S3). Finally, nine RCTs were included in the systematic review and meta-analysis (Figure 1).

### 3.2. Characteristics Summary of the Included Studies

Nine RCTs involving 856 patients were included in this review [11,12,13,14,15,16,17,18,19] (Table 1). All trials enrolled women with operable breast cancer undergoing MRM; one trial described the procedure as total mastectomy with level II axillary dissection [11], which corresponds to MRM as defined in this review. Across all trials, the intervention consisted of a single axillary drain (in one trial inserted beneath the lower flap and directed toward the axilla [17]), whereas the comparator consisted of two drains, with one placed in the axilla and the other beneath the pectoral/mastectomy flap or within the mastectomy bed. Detailed study designs, surgical and drain-management characteristics, seroma definitions, and baseline participant characteristics are summarized in Table 1, Table 2, Table 3 and Table 4.

The extent of axillary dissection was explicitly reported in only three trials (level II in two [11,16] and clearance to the apex of the axilla medial to pectoralis minor in one [13]), and no trial reported the use of flap fixation or quilting sutures (Table 2). All trials used closed-suction (negative-pressure) drains; the suction pressure (continuous negative pressure of 40 cm H_2_O) and the drain caliber (14-F polyethylene tubes) were specified in only one trial [18], and one further trial specified low-vacuum drains [11]. Drains were removed once the daily output fell below approximately 30 mL/24 h in eight trials, with two trials requiring the threshold on two consecutive days [11,12], two specifying a maximum of 10 postoperative days [14,15], and one removing the flap drain on postoperative day (POD) 5 and the axillary drain by output [12]; one trial used a weight-based threshold (< 0.5 mL/kg/24 h) with ultrasonographic confirmation [18]. In three trials, drain duration in the double-drain arm explicitly referred to the time until the last (axillary) drain was removed [12,15,17]; the remaining trials reported a single value per arm without specifying how it was derived.

Seroma definitions, assessment methods, and follow-up schedules varied across trials (Table 3). An explicit definition was provided in four trials: a symptomatic fluid collection beneath the flaps after drain removal within 30 days of surgery [16]; fluid accumulation below the flaps assessed clinically and by ultrasonography one day after drain removal [17]; symptomatic (Common Terminology Criteria for Adverse Events [CTCAE] grade 2–3) seroma requiring aspiration or intervention [11]; and seroma defined operationally by the need for needle aspiration [13]. The remaining trials detected seroma clinically (swelling or fluctuation) at scheduled visits, with ultrasonography used to confirm doubtful cases [19] or to confirm clinically observed collections 3–5 days after surgery and before drain removal [18]. Follow-up ranged from approximately 4 to 6 weeks and was not reported in three trials [12,18,19]. In all nine trials, the seroma counts entered into the meta-analysis represent clinically detected or symptomatic collections that were managed by needle aspiration rather than subclinical collections: in two trials, collections detected only by ultrasonography were reported separately and were not used, namely thin-film pectoral collections that almost all resolved without aspiration (5 vs. 1 patients) [18] and ultrasonographically detected seromas one day after drain removal (18/30 vs. 10/30) [17], for which the clinically detected outcome (13/30 vs. 8/30) was used instead. The denominators for the volume of aspirated seroma and the number of aspirations also differed: aspirated volume was reported across all randomized patients in two trials [13,15] and among patients with seroma in four [11,12,18,19], whereas the number of aspirations was reported across all patients in three trials [13,16,17] and among patients with seroma in two [11,12].

### 3.3. Summary of Risk of Bias and Certainty of Evidence

**Figure 2:**
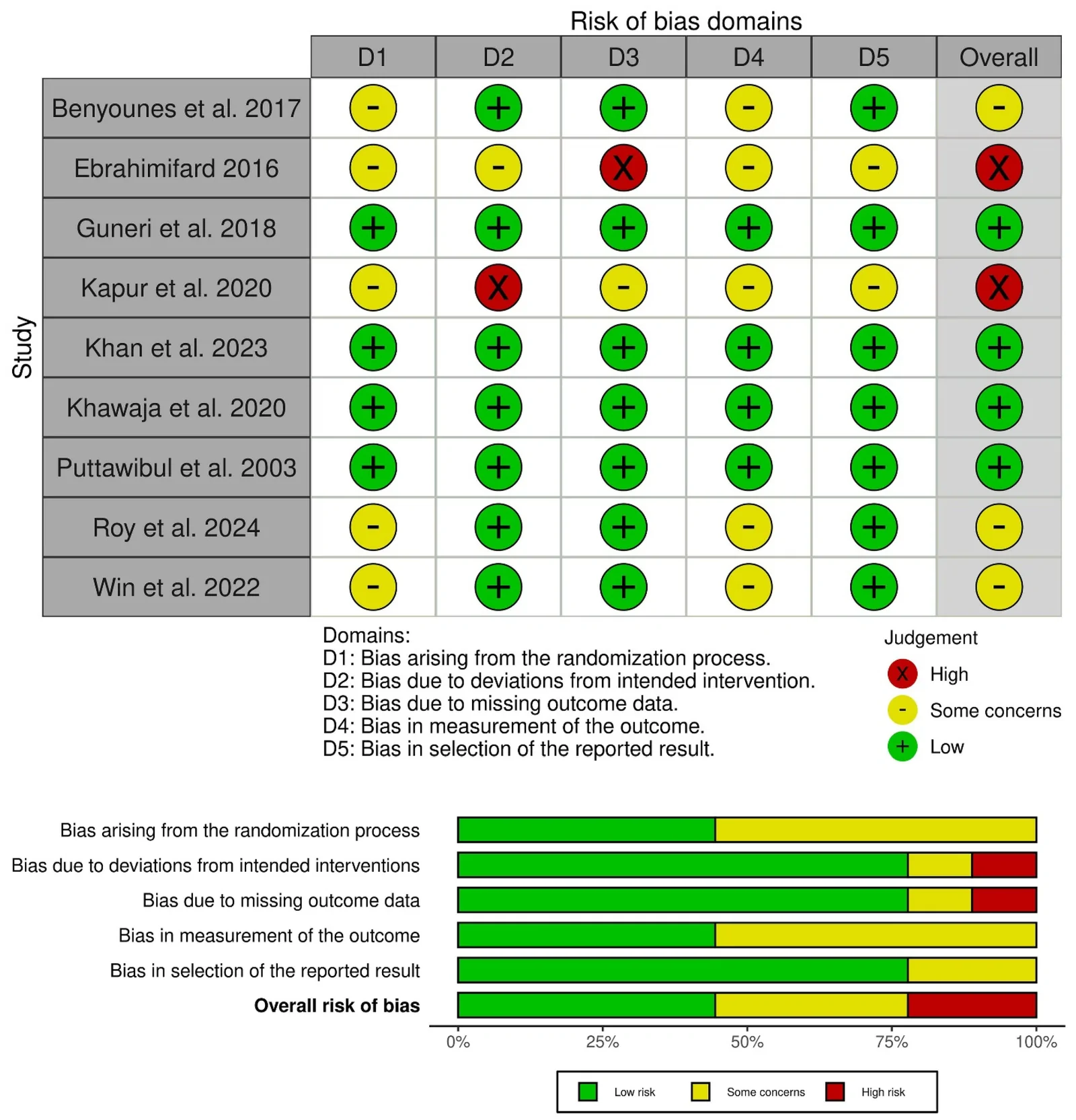
Quality assessment of risk of bias in the included randomized trials using the RoB 2 tool. Green indicates low risk, yellow indicates some concerns, and red indicates high risk of bias [11,12,13,14,15,16,17,18,19].

Among the nine included RCTs, four were judged to have a low overall risk of bias [16,17,18,19], three to have some concerns [11,12,14], and two to have a high overall risk of bias [13,15] (Figure 2). The GRADE evidence profile, including anticipated absolute effects, is presented in Table 5. The certainty of evidence was low for seroma formation, hematoma, SSI, total drain output, drain duration, and the number of seroma aspirations; very low for flap necrosis; and moderate for the volume of aspirated seroma. The pooled risk of seroma with double drainage was 234 per 1000 patients, and single drainage corresponded to an anticipated absolute difference of 30 more seromas per 1000 patients (95% CI: 33 fewer to 110 more). The corresponding anticipated absolute differences for hematoma, flap necrosis, and SSI were 13 more (95% CI: 6 fewer to 79 more), 14 fewer (95% CI: 33 fewer to 24 more), and 7 fewer (95% CI: 20 fewer to 33 more) per 1000 patients, respectively.

### 3.4. Primary Outcome: Seroma (%)

**Figure 3:**
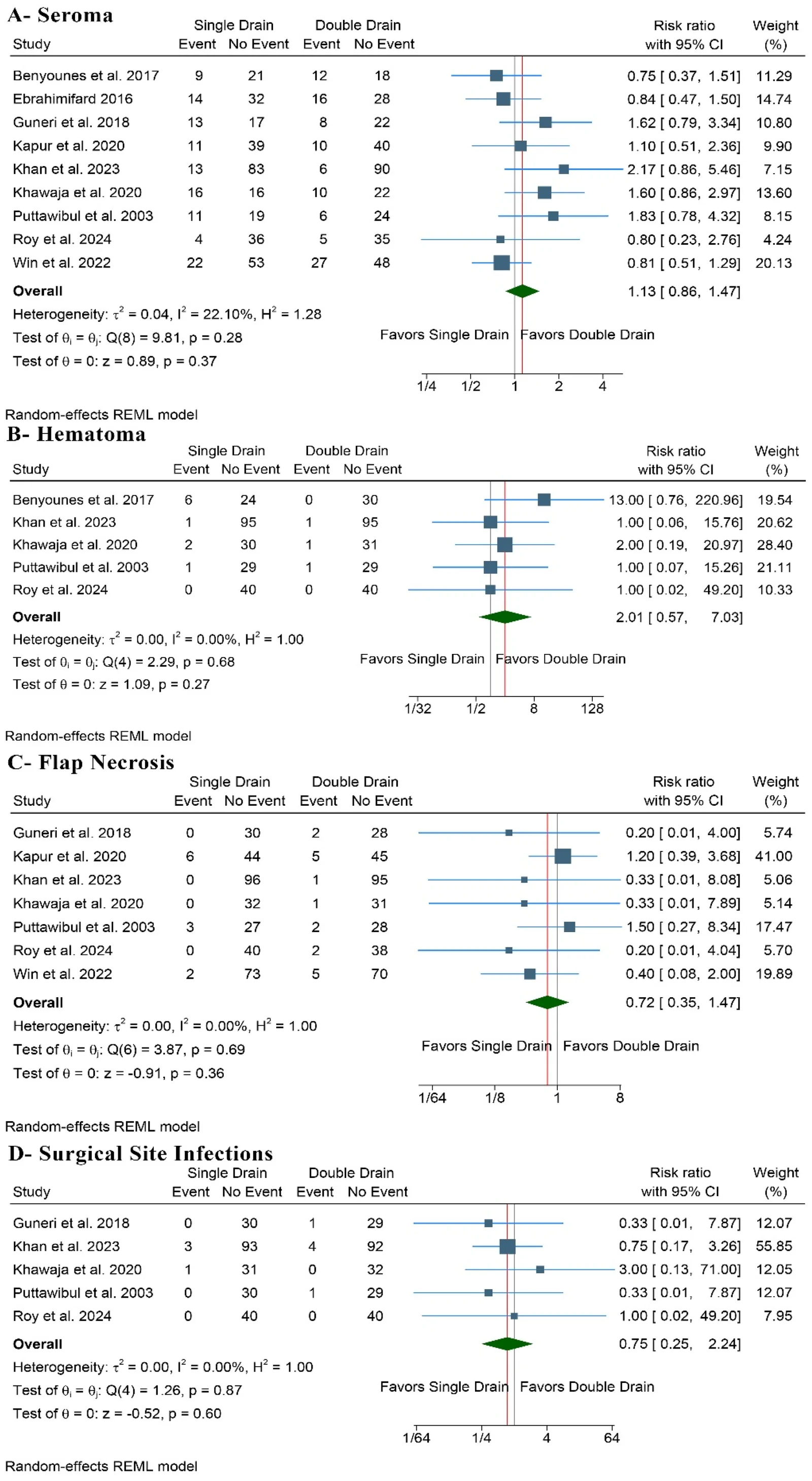
Forest plots of (**A**) Seroma, (**B**) Hematoma, (**C**) Flap Necrosis, and (**D**) Surgical Site Infection [11,12,13,14,15,16,17,18,19].

There was no significant difference between single-drain and double-drain placement in the incidence of postoperative seroma (RR: 1.13, 95% CI [0.86, 1.47], *p* = 0.37, I^2^ = 22.10%; low certainty) (Figure 3A). The overall pooled analyses were homogenous. The trial-level estimates did not differ systematically according to the seroma definition used (Table 3): RRs ranged from 0.81 to 2.17 in the three trials with symptomatic or aspiration-based definitions [11,13,16] and from 0.75 to 1.83 in the six trials that counted clinically detected (and aspirated) seromas [12,14,15,17,18,19], and the 95% CIs of all nine trials included the null.

### 3.5. Secondary Outcomes

#### 3.5.1. Postoperative Complications

##### Hematoma (%)

The incidence of postoperative hematoma did not differ significantly between the single-drain and double-drain groups (RR: 2.01, 95% CI [0.57, 7.03], *p* = 0.27, I^2^ = 0%; low certainty) (Figure 3B). The overall pooled analyses were homogenous. Only 13 events were reported in total; one trial reported no hematoma in either arm [12] and contributed an RR of 1.00 with a very wide CI (10.3% weight) after continuity correction, and the 95% CI of every individual trial included the null.

##### Flap Necrosis (%)

There was no significant difference between single-drain and double-drain placement in the incidence of flap necrosis (RR: 0.72, 95% CI [0.35, 1.47], *p* = 0.36, I^2^ = 0%; very low certainty) (Figure 3C). The overall pooled analyses were homogenous, but only 29 events were reported in total.

##### Surgical Site Infection (%)

Similarly, the incidence of SSI did not differ significantly between the single-drain and double-drain groups (RR: 0.75, 95% CI [0.25, 2.24], *p* = 0.60, I^2^ = 0%; low certainty) (Figure 3D). The overall pooled analyses were homogenous. Only 10 events were reported in total, and one trial with no events in either arm [12] contributed an RR of 1.00 (8.0% weight) after continuity correction.

### 3.6. Drainage-Related Outcomes

#### 3.6.1. Total Drain Output (mL)

**Figure 4:**
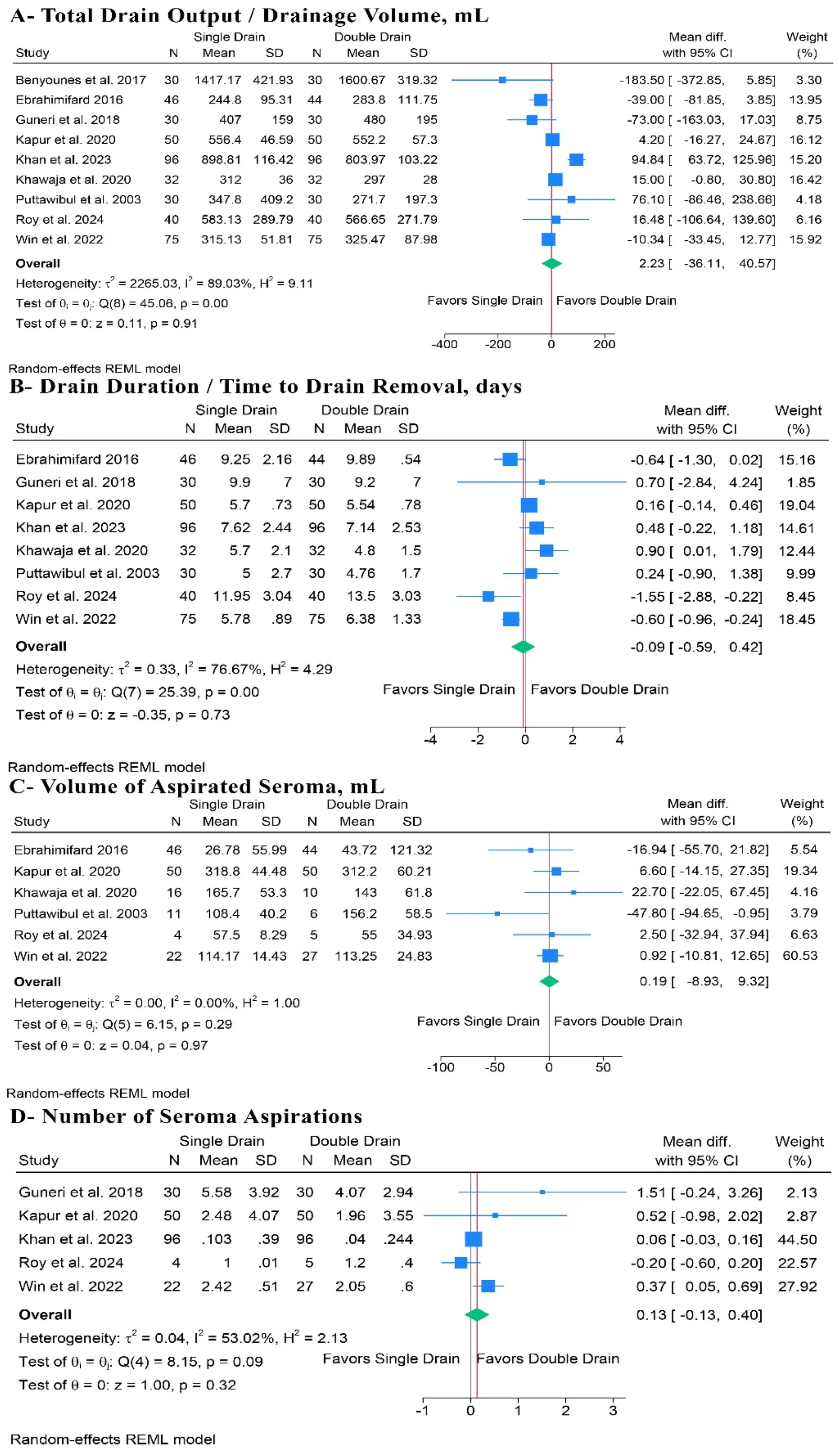
Forest plots of (**A**) Total Drain Output, (**B**) Drain Duration, (**C**) Volume of Aspirated Seroma, and (**D**) Number of Seroma Aspirations [11,12,13,14,15,16,17,18,19].

There was no significant difference between single-drain and double-drain placement in total postoperative drain output (MD: 2.23 mL, 95% CI [−36.11, 40.57], *p* = 0.91, I^2^ = 89.03%) (Figure 4A). Leave-one-out sensitivity analysis showed that the result remained statistically nonsignificant regardless of which trial was omitted (Figure S1). The Galbraith plot identified Khan et al. as a potential contributor to the observed heterogeneity [16] (Figure S2).

#### 3.6.2. Drain Duration (Days)

Drain duration did not differ significantly between the single-drain and double-drain groups (MD: −0.09 days, 95% CI [−0.59, 0.42], *p* = 0.73, I^2^ = 76.67%) (Figure 4B). The finding remained statistically nonsignificant in the leave-one-out sensitivity analysis regardless of the excluded trial (Figure S3). The Galbraith plot identified Khawaja et al. [19], Roy et al. [12], and Win et al. [11] as potential contributors to the observed heterogeneity (Figure S4).

#### 3.6.3. Volume of Aspirated Seroma (mL)

There was no significant difference between the two groups in the volume of seroma aspirated after drain removal (MD: 0.19 mL, 95% CI [−8.93, 9.32], *p* = 0.97, I^2^ = 0%) (Figure 4C). The overall pooled analyses were homogenous.

#### 3.6.4. Number of Seroma Aspirations

The number of post-removal seroma aspirations did not differ significantly between single-drain and double-drain placement (MD: 0.13 aspirations, 95% CI [−0.13, 0.40], *p* = 0.32, I^2^ = 53.02%) (Figure 4D). Leave-one-out sensitivity analysis showed that the pooled estimate remained statistically nonsignificant after omission of any individual trial (Figure S5). No individual study was clearly identified as an outlier on the Galbraith plot (Figure S6).

#### 3.6.5. Pain and Patient Comfort (Narrative Synthesis)

Pain or patient comfort was measured in only three trials, using different instruments and time points, which precluded pooling. Khan et al. assessed pain 24 h after surgery with a 0–10 visual analogue scale (VAS) and reported significantly lower scores with a single drain (mean ± SD: 2.5 ± 0.70 vs. 5.22 ± 5.10; *p* < 0.001), with a similar length of hospital stay (1.04 vs. 1.05 days) because most patients were discharged with drains in situ on POD 1 [16]. Guneri et al. used a non-validated 0–10 drain-related comfort scale and found no significant differences in pain around the drain (3.3 ± 1.7 vs. 3.7 ± 1.9; *p* = 0.42), discomfort attributed to the drain (3.1 ± 1.8 vs. 3.5 ± 2.1; *p* = 0.49), drain-related sleep disturbance (median 2 vs. 2; *p* = 0.31), or incisional pain (median 5 vs. 4; *p* = 0.18), with a median hospital stay of 3 days in both groups [17]. Kapur et al. reported VAS pain scores on postoperative days 0, 1, and 2 and one week after discharge that did not differ significantly between groups (for example, 46 vs. 55 on day 1 and 47.1 vs. 53.8 one week after discharge; scale range not specified; *p* = 0.10–0.20), an identical time to resumption of normal physical activity (5.54 ± 0.7 days in both groups), and similar rates of shoulder dysfunction (12% vs. 10%), numbness (4% vs. 4%), and lymphedema (8% vs. 6%) [13]. No trial measured patient satisfaction, quality of life, home-care burden, or costs.

## 4. Discussion

After synthesizing nine RCTs involving 856 patients, the main finding was that single-drain placement was not associated with a statistically significant increase in postoperative seroma formation. Similarly, no significant differences were observed in hematoma, flap necrosis, surgical-site infection, total drain output, drain duration, volume of aspirated seroma, or the number of post-removal seroma aspirations. These findings were generally consistent across clinical complication outcomes, with little to no heterogeneity. In contrast, significant heterogeneity was observed for total drain output and drain duration. The certainty of evidence was low for most outcomes (very low for flap necrosis and moderate only for the volume of aspirated seroma), and the 95% CI for seroma was compatible with effects ranging from 33 fewer to 110 more seromas per 1000 patients. Therefore, the available randomized evidence does not demonstrate a clear clinical advantage from routinely adding a pectoral or sub-flap drain to an axillary drain after MRM, but it also cannot exclude a clinically relevant increase in seroma with single drainage.

Seroma is a multifactorial process, not a simple consequence of inadequate fluid evacuation. Disruption of lymphatic vessels during axillary dissection, inflammatory exudation, and separation of the skin flaps from the chest wall may all contribute to seroma formation [5,32,33]. Previous studies have also explored patient- and procedure-related factors, including BMI, breast size, extent of axillary dissection, diabetes, age, and surgical technique; however, associations have not been consistent across studies [4,5,32,34]. Our pooled results did not show a significant reduction in seroma formation with double drainage, with low heterogeneity. Hence, the assumption that the mastectomy bed necessarily requires an additional pectoral drain to prevent seroma is not supported by the pooled randomized evidence. A plausible explanation is that an axillary drain may be sufficient to evacuate freely communicating postoperative fluid, whereas continued lymphatic leakage and failure of flap adherence are not necessarily corrected by adding another drainage catheter.

The findings for seroma after drain removal further support this interpretation. Similar overall seroma rates could theoretically conceal a greater postoperative burden in patients treated with a single drain if the resulting collections were larger or required more frequent aspiration. But this was not the case in our analysis. The mean difference in aspirated seroma volume was only 0.19 mL, with a narrow confidence interval around the null and no observed heterogeneity. Likewise, the number of seroma aspirations did not differ significantly between strategies, with a pooled difference of only 0.13 aspirations. Although moderate heterogeneity was present for the number of aspirations, leave-one-out analysis showed that the pooled finding remained nonsignificant after exclusion of every individual study. This is relevant because clinically significant seroma requiring repeated aspiration arguably represents a more patient-important outcome than the mere detection of a postoperative fluid collection. Repeated aspirations can increase discomfort, necessitate additional clinic visits, and expose the wound to repeated instrumentation [5,6,33].

Differences in how seroma was defined and ascertained nevertheless deserve emphasis (Table 3). Ultrasonographically detected collections, clinically palpable collections, and symptomatic seromas requiring intervention are not interchangeable, and their incidence can differ substantially within the same trial: in the trial by Guneri et al., ultrasonography one day after drain removal detected seroma in 60% versus 33% of patients, compared with clinical rates of 43% versus 27% [17]. We therefore examined the definitions used by each trial. In all nine trials, the counted seromas were either defined as symptomatic collections requiring aspiration or were clinically detected collections that were all managed by aspiration, whereas collections detected only by ultrasonography in two trials were deliberately not used, so that the pooled estimate reflects clinically relevant seroma; a separate analysis restricted to seromas requiring aspiration would therefore have reproduced the primary analysis with the same data and was not performed. Reassuringly, the trial-level estimates did not differ systematically between definitions, statistical heterogeneity was low, and the number of post-removal aspirations and the aspirated volume, which directly reflect seromas requiring intervention, did not differ between strategies. Nevertheless, the absence of a standardized definition, the variable assessment schedules (from a scheduled ultrasonographic assessment at 3–5 days followed by clinical review of unreported duration, to clinical review for up to 6 weeks), the incomplete reporting of follow-up in three trials, and the mixed denominators used for the aspiration outcomes remain sources of clinical heterogeneity that statistical tests cannot fully capture.

Moreover, total drain output and drain duration require careful consideration, as they were the most heterogeneous outcomes. Nevertheless, the leave-one-out analyses remained statistically nonsignificant regardless of which study was omitted, indicating that the overall absence of a detectable difference was not dependent on a single trial. These differences are clinically plausible because the included studies did not use standardized drainage protocols. Removal criteria ranged from fixed postoperative-day approaches to output thresholds, typically around 30 mL/day, with some studies requiring the threshold to be reached on consecutive days [11,12,13,14,15,16,17,18,19]. Suction systems and drain positioning also differed, the extent of axillary dissection was reported in only three trials, and no trial used flap fixation or quilting, so that our findings apply to conventional wound closure without dead-space obliteration (Table 2). In addition, the meaning of drain duration in the double-drain arm was explicitly defined as the time until the last drain was removed in only three trials [12,15,17]; in the others, it is unclear whether the reported value referred to the last drain or to an average across drains, which may have attenuated or exaggerated the between-arm differences. Similar variability has been described throughout the breast surgery literature. Trials evaluating early versus later drain removal have shown that keeping drains in place longer does not consistently prevent subsequent seroma formation [35,36,37]. Also, randomized evidence comparing different suction pressure levels indicates that the characteristics of the drainage system itself can affect postoperative drainage patterns [38]. Therefore, the observed significant heterogeneity likely reflects differences in perioperative and drain-management protocols rather than a consistent effect attributable only to the number of drains.

This review has several strengths. It focused exclusively on RCTs directly comparing single with double drainage, thereby minimizing confounding associated with surgeon preference, patient selection, and differences in operative complexity. Nine randomized trials and 856 patients were included, providing a larger evidence base than any individual trial and, to our knowledge, the first quantitative synthesis of this comparison. Risk of bias was evaluated using RoB 2, and the certainty of each outcome was formally evaluated using GRADE with explicit imprecision thresholds and anticipated absolute effects. Random-effects models were used because clinical heterogeneity was anticipated, and leave-one-out sensitivity analyses and Galbraith plots were applied to outcomes with substantial heterogeneity. 

Several limitations should still be considered. First, the methodological quality of the included evidence was variable. Only four trials were judged to have a low overall risk of bias, three had some concerns, and two were at high risk of bias. This directly contributed to the downgrading of the certainty of several outcomes. Second, many of the trials were relatively small, and the numbers of events for hematoma, flap necrosis, and surgical-site infection were low, resulting in wide confidence intervals and a limited ability to exclude clinically important differences; the pooled sample size for seroma was also below the optimal information size, and the 95% CI was compatible with a clinically relevant increase in seroma with single drainage. Trials with zero events in both arms were retained with a continuity correction (the default in Stata), which pulls the pooled estimate slightly toward the null; given the very small number of events, their exclusion would not have materially changed the pooled estimates, which were already imprecise, or their interpretation. Third, substantial heterogeneity was present for total drain output and drain duration. Although sensitivity analyses showed that the conclusions remained stable, heterogeneity in drain positioning, suction systems, surgical techniques, drain-removal thresholds, and postoperative protocols reduces the precision with which these pooled estimates can be translated into a single clinical protocol. Fourth, definitions and assessment methods for seroma were not standardized, follow-up was not reported in three trials, and the denominators for the aspiration outcomes differed between trials (Table 3). Fifth, potentially important patient-level determinants such as BMI, diabetes, breast size, neoadjuvant systemic therapy, extent of nodal dissection, and smoking were inconsistently reported, and could not be explored through reliable analyses. Sixth, outcomes directly relevant to the proposed advantages of using fewer drains, including pain, patient comfort, mobility, satisfaction, length of hospital stay, home-care burden, and healthcare costs, were insufficiently and inconsistently reported. Seventh, the full text of one potentially eligible randomized trial published in 1992 could not be retrieved [25]; its abstract reported no significant difference in overall complications between one and two drains (35.0% vs. 31.9%) but did not provide extractable outcome data by group, and we cannot exclude the existence of additional eligible reports in regional or non-English-language journals that are not indexed in the databases searched. Finally, publication bias and small-study effects could also not be formally assessed because fewer than ten studies contributed to each pooled outcome.

From a clinical perspective, these findings indicate that the routine addition of a second pectoral or sub-flap drain has not been shown to reduce seroma or other complications in randomized trials; they do not, however, establish that single drainage is equivalent or non-inferior, because the 95% CI for seroma was compatible with up to 110 additional seromas per 1000 patients and the certainty of evidence was low. A single-drain strategy has several potential practical advantages, including fewer drain exit sites, simpler postoperative care, easier mobility, and less drain-related discomfort. These potential benefits are relevant because current perioperative breast care increasingly emphasizes early mobilization, reduced treatment burden, and timely discharge [39,40]. Nevertheless, the direct evidence for these advantages is limited: pain or comfort was measured in only three trials, with less pain at 24 h with a single drain in one trial [16] and no difference in the other two [13,17], and no trial measured satisfaction, quality of life, or costs. These advantages were therefore not reported consistently enough across the included trials to permit pooled assessment, and they should remain considered potential rather than established benefits. 

Drain practice also remains heterogeneous in current clinical care; a national United Kingdom survey demonstrated substantial variation in the use of drains and in the management of seroma after mastectomy and axillary surgery [41]. Enhanced-recovery approaches to breast surgery similarly support minimizing unnecessary postoperative interventions while maintaining safety and patient-centered recovery [39,42]. Accordingly, the present evidence does not support the view that a second pectoral or sub-flap drain is obligatory, but neither does it justify abandoning it in all patients; decisions should account for operative findings, extent of axillary surgery, flap size, dead space, bleeding, reconstructive procedures, and other patient-specific factors. Finally, the evidence in this review applies to women undergoing MRM (total mastectomy with ALND) with conventional wound closure and should not automatically be applied to immediate reconstruction, nipple-sparing procedures, flap fixation or quilting techniques, or other contemporary breast operations with different anatomical and postoperative considerations.

Future trials should therefore move beyond simply asking whether one or two drains result in a statistically different seroma rate. Patient-centered outcomes should receive greater attention, including postoperative pain, interference with sleep and mobility, ease of home drain care, return to normal activity, satisfaction, unscheduled clinic visits, and total healthcare costs, and seroma should be defined and ascertained in a standardized manner (for example, as a symptomatic collection requiring aspiration, assessed at fixed time points). Future studies should also examine whether treatment effects differ in patients at higher baseline risk of seroma, including those with obesity, diabetes, larger mastectomy flaps, extensive axillary surgery, or neoadjuvant treatment [43]. An individual-patient-data approach may ultimately be useful for identifying patients in whom the second drain provides meaningful benefit rather than applying the same strategy to all patients. In addition, the drain number should be studied alongside modern methods of dead-space reduction. Growing evidence supporting flap fixation and quilting suggests that the optimal postoperative strategy may involve an interaction between wound closure technique and drain requirement, rather than the drain number alone [44,45,46]; a randomized trial comparing one versus two drains in patients undergoing MRM with flap quilting in both arms is currently recruiting (NCT07512141).

## 5. Conclusions

Single axillary drainage after MRM was not associated with statistically significant differences in postoperative seroma formation, aspirated seroma volume, number of seroma aspirations, total drain output, drain duration, hematoma, flap necrosis, or surgical-site infection compared with double axillary plus pectoral/sub-flap drainage. Accordingly, the available evidence does not demonstrate a clear clinical advantage from routinely placing a second drain. However, the certainty of evidence was low for most outcomes, and the pooled estimate for seroma was compatible with both a small reduction and a clinically relevant increase, so the two strategies cannot be considered equivalent on the basis of the current evidence. A single-drain strategy may therefore be reasonable in selected patients and could reduce drain-related burden, but adequately powered trials with standardized seroma definitions, defined drain-management protocols, and patient-centered outcomes are needed to determine whether specific patient or operative characteristics identify subgroups that benefit from additional drainage.

## Figures and Tables

**Table 1:** Characteristics of the included randomized trials.

Study ID (Country)	Design	Participants, n (Single/Double Drain)	Main Inclusion Criteria	Primary Outcome	Follow-Up Duration
Benyounes et al., 2017 (Egypt) [14]	Parallel-group RCT	30/30	Female patients with breast cancer, mainly stage I–II, undergoing MRM	Seroma formation	4 weeks (weekly after drain removal)
Ebrahimifard 2016 (Iran) [15]	Parallel-group RCT	46/44	Female patients with invasive breast cancer unsuitable for breast-conserving surgery, undergoing MRM	Seroma formation	4 weeks (weekly after drain removal)
Guneri et al., 2018 (Turkey) [17]	Parallel-group RCT	30/30	Female patients aged 18–80 years with breast carcinoma undergoing MRM (patients with a negative sentinel lymph node biopsy excluded)	Patient comfort and seroma formation	Up to 4 weeks
Kapur et al., 2020 (India) [13]	Parallel-group RCT	50/50	Female patients with operable breast carcinoma undergoing MRM	Seroma formation	4 weeks after drain removal
Khan et al., 2023 (Pakistan) [16]	Parallel-group RCT	96/96 (98/98 randomized)	Female patients with biopsy-proven breast carcinoma undergoing MRM (immediate reconstruction excluded)	Seroma formation	30 days
Khawaja et al., 2020 (Pakistan) [19]	Parallel-group RCT	32/32	Female patients with breast carcinoma confirmed by FNAC or Tru-cut biopsy, undergoing MRM	Seroma formation	NR
Puttawibul et al., 2003 (Thailand) [18]	Parallel-group RCT	30/30	Female patients aged 35–70 years with primary breast cancer undergoing MRM (insulin-dependent diabetes, hypertension, anticoagulant or steroid therapy, preoperative radiotherapy or chemotherapy, and T4 tumors excluded)	Total drainage volume and postoperative complications (seroma)	NR
Roy et al., 2024 (India) [12]	Parallel-group RCT	40/40	Female patients with breast carcinoma undergoing upfront MRM or MRM after neoadjuvant systemic therapy	Seroma formation	NR
Win et al., 2022 (Myanmar) [11]	Parallel-group RCT	75/75	Female patients with stage I–II breast cancer undergoing total mastectomy with level II axillary dissection (i.e., MRM)	Incidence of grade 2–3 seroma	6 weeks

Abbreviations: FNAC, fine-needle aspiration cytology; MRM, modified radical mastectomy; NR, not reported; RCT, randomized controlled trial.

**Table 2:** Surgical technique and drain management in the included trials.

Study ID	Surgical Procedure and Extent of Axillary Dissection	Flap Fixation or Quilting	Drain Type and Suction System	Single-Drain Arm	Double-Drain Arm	Drain Removal Criteria	Definition of Drain Duration in the Double-Drain Arm
Benyounes et al., 2017 [14]	MRM; extent of ALND NR; axillary dissection by scissors (45%) or diathermy (55%)	NR	Closed-suction drains; caliber and suction pressure NR	One axillary drain	One axillary + one pectoral drain	Output < 30 mL/24 h or POD 10 (patients discharged with drains after 48 h)	Not specified (one value per patient; not included in the pooled drain-duration analysis)
Ebrahimifard 2016 [15]	MRM (Madden technique); extent of ALND NR	NR	Closed-suction drains; caliber and suction pressure NR	One axillary drain	One axillary + one pectoral (sub-flap) drain	Output < 30 mL/24 h or POD 10 (patients discharged with drains after 48 h)	Each drain reported separately; pectoral drain removed at 2.9 ± 0.9 days; duration of the axillary (last) drain used
Guneri et al., 2018 [17]	MRM with axillary dissection; extent of ALND NR	NR	Negative-pressure drains; caliber and suction pressure NR	One drain inserted below the lower flap and directed toward the axilla	One axillary + one sub-flap drain	Output < 30 mL/24 h (patients could be discharged with drains)	Duration of the longer-staying (last) drain
Kapur et al., 2020 [13]	MRM with diathermy; axillary clearance up to the apex of the axilla medial to pectoralis minor (pectoralis minor preserved)	NR	Closed-suction drains; caliber and suction pressure NR	One axillary drain	One axillary + one pectoral drain	Output < 30 mL/24 h	Not specified (one value per arm)
Khan et al., 2023 [16]	MRM; standard level II axillary clearance; pectoralis major and minor retained; electrocautery	NR (skin closed with staples)	Closed-suction (vacuum-bottle) drains; caliber and suction pressure NR	One axillary drain	One axillary + one sub-flap drain	Output < 30 mL/24 h (most patients discharged with drains on POD 1)	Not specified (one value per arm)
Khawaja et al., 2020 [19]	MRM; flaps raised with scalpel and axilla dissected with scissors (no electrocautery); extent of ALND NR	NR	Suction drains of uniform gauge with constant negative pressure (values NR)	One axillary drain	One axillary + one flap/pectoral drain	Output ≤ 30 mL/24 h	Not specified (one value per arm)
Puttawibul et al., 2003 [18]	MRM; mastectomy by scalpel with cautery of individual bleeding points, axillary dissection with scissors and silk ligation; extent of ALND NR	NR	14-F polyethylene tube drains with continuous negative suction of 40 cm H_2_O	One axillary drain	One axillary + one pectoral drain	Kept ≥ 2 days; removed when output < 0.5 mL/kg/24 h and ultrasonography showed no seroma (dressing opened on POD 3)	Not specified (one value per arm)
Roy et al., 2024 [12]	MRM (level I–II ALND per the authors’ definition); monopolar cautery for flaps and axilla, vessel sealer for larger vessels	NR (skin closed with silk sutures)	Closed-suction drains; caliber and suction pressure NR	One axillary drain	One axillary + one mastectomy-flap drain	Flap drain removed on POD 5; axillary drain removed when output < 30 mL/24 h for 2 consecutive days	Each drain reported separately; duration of the axillary (last) drain used
Win et al., 2022 [11]	Total mastectomy with level II axillary dissection (MRM); electrocautery; standardized technique by five trained surgeons	NR (axillary padding for 48 h in both groups)	Low-vacuum drains; caliber NR	One low-vacuum axillary drain	One axillary + one mastectomy-bed drain	Output < 30 mL/24 h for 2 consecutive days	Not specified (one value per arm)

Abbreviations: ALND, axillary lymph node dissection; F, French; MRM, modified radical mastectomy; NR, not reported; POD, postoperative day.

**Table 3:** Definition, assessment, and follow-up of seroma in the included trials.

Study ID	Seroma Definition	Method of Assessment	Assessment Schedule	Follow-Up Duration	Indication for Aspiration	Seroma Counts Used in the Meta-Analysis	Denominator for Aspirated Volume/Number of Aspirations
Benyounes et al., 2017 [14]	No explicit definition	Clinical detection at follow-up visits	Weekly for 1 month after removal of the last drain; every other day if seroma present	1 month	All detected seromas aspirated (no volume threshold)	Any clinically detected seroma (all aspirated)	NR (not included in these analyses)
Ebrahimifard 2016 [15]	No explicit definition	Clinical detection by the surgeon	Visits on days 2 and 10, then weekly for 1 month after removal of the last drain	1 month	All detected seromas aspirated (no volume threshold)	Any clinically detected seroma (all aspirated)	Aspirated volume: all randomized patients (46/44)
Guneri et al., 2018 [17]	Fluid accumulation below the flaps	Daily clinical examination until discharge; ultrasonography 1 day after drain removal; wound inspection after discharge	Daily until discharge; ultrasonography 1 day after drain removal; review up to 4 weeks	Up to 4 weeks	Aspiration performed when seroma present (no volume threshold)	Clinically detected seroma (13/30 vs. 8/30); ultrasonographically detected seroma (18/30 vs. 10/30) reported separately and not used	Number of aspirations: all randomized patients (30/30)
Kapur et al., 2020 [13]	Defined operationally by the need for needle aspiration	Clinical examination with needle aspiration	Days 1, 3, and 5 and 4 weeks after drain removal	4 weeks after drain removal	Seroma requiring needle aspiration	Seroma requiring aspiration	All randomized patients as reported (50/50)
Khan et al., 2023 [16]	Fluid collection beneath the skin flaps after removal of the drains causing patient discomfort within 30 days of surgery	Clinical assessment at outpatient follow-up	Outpatient visits within 30 days (schedule not specified)	30 days	Symptomatic seroma (aspiration performed; no volume threshold)	Symptomatic seroma within 30 days	Number of aspirations (punctures): all analyzed patients (96/96)
Khawaja et al., 2020 [19]	No explicit definition	Clinical examination; ultrasonography if clinically suspected	Dressing opened on POD 3; outpatient review at 1 week and thereafter (schedule not specified)	NR	Detected seromas aspirated (no volume threshold)	Any clinically detected seroma (axillary or pectoral)	Aspirated volume: patients with seroma (16/10)
Puttawibul et al., 2003 [18]	No explicit definition (loculated serous collection)	Clinical observation confirmed by ultrasonography; ultrasonography also used to guide aspiration (size and location)	Dressing opened on POD 3; ultrasonography 3–5 days after surgery and before drain removal; clinical follow-up (schedule not specified)	NR	Loculated axillary seroma aspirated (aspiration considered mandatory); thin-film pectoral collections seen only on ultrasonography were not aspirated	Axillary seroma requiring aspiration (11/30 vs. 6/30); pectoral ultrasonography-only collections (5 vs. 1) reported separately and not used	Aspirated volume: patients with axillary seroma (11/6)
Roy et al., 2024 [12]	No explicit definition (collection of serous fluid in the dead space)	Clinical wound examination; ultrasound-guided needle aspiration	Follow-up visits (schedule not specified)	NR	Detected seromas aspirated (no volume threshold)	Any clinically detected seroma (all aspirated)	Patients with seroma (4/5)
Win et al., 2022 [11]	CTCAE v3.0 grading; primary outcome: grade 2–3 (symptomatic) seroma requiring aspiration or intervention	History and clinical examination (swelling, fluctuation); ultrasonography and diagnostic aspiration in doubtful cases	Weeks 2, 3, 4, and 6	6 weeks	Symptomatic seroma aspirated until resolution (no volume threshold)	Symptomatic (grade 2–3) seroma	Patients with seroma (22/27)

Abbreviations: CTCAE, Common Terminology Criteria for Adverse Events; NR, not reported; POD, postoperative day. Numbers in parentheses are the group sizes (single/double drain) used in the pooled analyses of the volume of aspirated seroma and the number of aspirations.

**Table 4:** Baseline characteristics of the participants.

Study ID	Group	N	Age (Years)	BMI (kg/m^2^)	DM, n (%)	Smoking, n (%)	Operation Time (min)	Dissected Lymph Nodes, n	Tumor Size or Weight	Blood Loss (mL)
Benyounes et al., 2017 [14]	Single	30	53.83 (11.00)	29.62 (5.17)	NR	NR	99.60 (22.80)	12.10 (3.06)	NR	<200 mL: 10 (33.3%); 200–300 mL: 15 (50.0%); >300 mL: 5 (16.7%)
	Double	30	53.63 (9.37)	30.06 (4.14)	NR	NR	108.00 (22.80)	12.73 (2.78)	NR	<200 mL: 15 (50.0%); 200–300 mL: 13 (43.3%); >300 mL: 2 (6.7%)
Ebrahimifard 2016 [15]	Single	46	53.59 (14.45)	25.92 (3.58)	NR	NR	NR	NR	354.8 g (207.1)	NR
	Double	44	54.23 (12.17)	26.65 (3.64)	NR	NR	NR	NR	402.4 g (238.6)	NR
Guneri et al., 2018 [17]	Single	30	54.10 (11.60)	26.20 (4.80)	NR	9 (30.0)	NR	21.20 (5.70)	40.25 mm (20.75)	NR
	Double	30	53.90 (11.50)	26.90 (5.00)	NR	10 (33.3)	NR	19.50 (7.80)	36.75 mm (21.25)	NR
Kapur et al., 2020 [13]	Single	50	47.54 (8.30)	NR	NR	NR	NR	NR	NR	NR
	Double	50	45.86 (9.30)	NR	NR	NR	NR	NR	NR	NR
Khan et al., 2023 [16]	Single	96	55.13 (9.00)	23.21 (3.50)	20 (20.8) ^a^	0 (0)	103.97 (15.93)	15.43 (4.11)	NR	101.24 (25.15)
	Double	96	55.37 (8.40)	23.21 (3.20)	16 (16.7) ^a^	0 (0)	103.19 (15.96)	16.23 (4.01)	NR	101.67 (24.40)
Khawaja et al., 2020 [19]	Single	32	49.00 (13.20)	NR	NR	NR	NR	NR	NR	NR
	Double	32	47.00 (9.20)	NR	NR	NR	NR	NR	NR	NR
Puttawibul et al., 2003 [18]	Single	30	46.70 (11.10)	NR	NR	NR	106.10 (23.10)	NR	NR	315.70 (182.60)
	Double	30	45.00 (8.70)	NR	NR	NR	116.20 (38.70)	NR	NR	361.70 (157.40)
Roy et al., 2024 [12]	Single	40	46.58 (10.64)	24.19 (5.09)	NR	NR	NR	NR	533.98 g (199.99)	NR
	Double	40	52.58 (13.21)	24.72 (5.97)	NR	NR	NR	NR	631.58 g (306.58)	NR
Win et al., 2022 [11]	Single	75	48.66 (10.44)	28.20 (3.48)	NR	NR	NR	NR	NR	NR
	Double	75	51.22 (11.04)	28.79 (3.11)	NR	NR	NR	NR	NR	NR

Values are mean (SD) unless stated otherwise. N refers to the number of participants analyzed in each group. ^a^ Percentages recalculated using the number of patients analyzed (n = 96 per group) because the denominators printed in the original report were inconsistent. Abbreviations: BMI, body mass index; DM, diabetes mellitus; N, number; NR, not reported; SD, standard deviation.

**Table 5:** GRADE evidence profile with anticipated absolute effects.

Outcome	Participants (Studies)	Risk of Bias	Inconsistency	Indirectness	Imprecision	Publication Bias	Relative Effect (95% CI)	Risk with Double Drainage (per 1000)	Risk Difference with Single Drainage (per 1000; 95% CI)	Certainty of Evidence
Seroma	856 (9 RCTs)	Serious ^a^	Not serious	Not serious	Serious ^b^	Not assessed ^c^	RR 1.13 (0.86 to 1.47)	234	30 more (33 fewer to 110 more)	⊕⊕◯◯ Low
Hematoma	456 (5 RCTs)	Not serious	Not serious	Not serious	Very serious ^d^	Not assessed ^c^	RR 2.01 (0.57 to 7.03)	13	13 more (6 fewer to 79 more)	⊕⊕◯◯ Low
Flap necrosis	706 (7 RCTs)	Serious ^a^	Not serious	Not serious	Very serious ^d^	Not assessed ^c^	RR 0.72 (0.35 to 1.47)	51	14 fewer (33 fewer to 24 more)	⊕◯◯◯ Very low
Surgical-site infection	456 (5 RCTs)	Not serious	Not serious	Not serious	Very serious ^d^	Not assessed ^c^	RR 0.75 (0.25 to 2.24)	26	7 fewer (20 fewer to 33 more)	⊕⊕◯◯ Low
Total drain output or drainage volume (mL)	856 (9 RCTs)	Serious ^a^	Serious ^e^	Not serious	Not serious ^f^	Not assessed ^c^	—	Mean 271.7 to 1600.7 mL across trials	MD 2.23 mL higher (36.11 lower to 40.57 higher)	⊕⊕◯◯ Low
Drain duration or time to drain removal (days)	796 (8 RCTs)	Serious ^a^	Serious ^e^	Not serious	Not serious ^f^	Not assessed ^c^	—	Mean 4.8 to 13.5 days across trials	MD 0.09 days lower (0.59 lower to 0.42 higher)	⊕⊕◯◯ Low
Volume of aspirated seroma (mL)	291 (6 RCTs) ^g^	Serious ^a^	Not serious	Not serious	Not serious ^f^	Not assessed ^c^	—	Mean 43.7 to 312.2 mL across trials	MD 0.19 mL higher (8.93 lower to 9.32 higher)	⊕⊕⊕◯ Moderate
Number of seroma aspirations	410 (5 RCTs) ^g^	Serious ^a^	Serious ^e^	Not serious	Not serious ^f^	Not assessed ^c^	—	Mean 0.04 to 4.07 across trials	MD 0.13 aspirations higher (0.13 lower to 0.40 higher)	⊕⊕◯◯ Low

The assumed risk with double drainage is the pooled control-group risk across the trials contributing to each outcome (seroma: 100/427; hematoma: 3/228; flap necrosis: 18/353; surgical-site infection: 6/228). ^a^ Downgraded one level because trials at high overall risk of bias (Ebrahimifard 2016 [15]; Kapur et al., 2020 [13]) and/or trials with some concerns contributed substantial weight to the pooled estimate. ^b^ Downgraded one level because the 95% CI crossed the threshold of appreciable harm (RR 1.25) and the pooled sample size (856 participants; 213 events) was below the optimal information size (approximately 1480 participants to detect a 25% relative risk reduction from a control-group risk of 23.4% with 80% power and α = 0.05). ^c^ Publication bias was not formally assessed because fewer than 10 trials contributed to each outcome. ^d^ Downgraded two levels because of very few events (13, 29, and 10 events for hematoma, flap necrosis, and surgical-site infection, respectively) and 95% CIs that included both appreciable benefit and appreciable harm (RR < 0.75 and >1.25). ^e^ Downgraded one level for substantial statistical heterogeneity (I^2^ = 89%, 77%, and 53% for total drain output, drain duration, and the number of aspirations, respectively), attributable to differences in drain-management protocols. ^f^ Not downgraded because the 95% CI lay entirely within the thresholds for a clinically important difference specified in the Methods (100 mL for total drain output; 1 day for drain duration; 50 mL for aspirated volume; 1 aspiration). ^g^ Number of participants analyzed; the denominators were all randomized patients in some trials and patients who developed seroma in others (Table 3). Abbreviations: CI, confidence interval; GRADE, Grading of Recommendations Assessment, Development and Evaluation; MD, mean difference; RCT, randomized controlled trial; RR, risk ratio.

## Data Availability

The study-level data underlying all meta-analyses (numbers of events and participants, and means, standard deviations, and sample sizes for each trial and outcome) are presented in full in the forest plots (Figure 3 and Figure 4). The data extraction sheet and the Stata analysis commands (do-file) are available from the corresponding author upon reasonable request.

## Supplementary Materials

The following supporting information can be downloaded at: https://www.mdpi.com/article/10.3390/jcm15218391/s1, Table S1: The PRISMA 2020 checklist; Table S2: Search strategy; Table S3: Excluded records in full-text screening; Figure S1: Leave-one-out sensitivity analysis for total drain output; Figure S2: Galbraith plot for total drain output; Figure S3: Leave-one-out sensitivity analysis for drain duration; Figure S4: Galbraith plot for drain duration; Figure S5: Leave-one-out sensitivity analysis for the number of seroma aspirations; Figure S6: Galbraith plot for the number of seroma aspirations.
